# Approaches to Dispersing Medical Biofilms

**DOI:** 10.3390/microorganisms5020015

**Published:** 2017-04-01

**Authors:** Derek Fleming, Kendra P. Rumbaugh

**Affiliations:** Departments of Surgery, Immunology and Molecular Microbiology, and the TTUHSC Burn Center of Research Excellence, Texas Tech University Health Sciences Center, Lubbock, TX 79430, USA; derek.fleming@ttuhsc.edu

**Keywords:** biofilm, dispersal, dispersal agent

## Abstract

Biofilm-associated infections pose a complex problem to the medical community, in that residence within the protection of a biofilm affords pathogens greatly increased tolerances to antibiotics and antimicrobials, as well as protection from the host immune response. This results in highly recalcitrant, chronic infections and high rates of morbidity and mortality. Since as much as 80% of human bacterial infections are biofilm-associated, many researchers have begun investigating therapies that specifically target the biofilm architecture, thereby dispersing the microbial cells into their more vulnerable, planktonic mode of life. This review addresses the current state of research into medical biofilm dispersal. We focus on three major classes of dispersal agents: enzymes (including proteases, deoxyribonucleases, and glycoside hydrolases), antibiofilm peptides, and dispersal molecules (including dispersal signals, anti-matrix molecules, and sequestration molecules). Throughout our discussion, we provide detailed lists and summaries of some of the most prominent and extensively researched dispersal agents that have shown promise against the biofilms of clinically relevant pathogens, and we catalog which specific microorganisms they have been shown to be effective against. Lastly, we discuss some of the main hurdles to development of biofilm dispersal agents, and contemplate what needs to be done to overcome them.

## 1. Introduction

Biofilms are communities of microorganisms protected by a self-synthesized layer of complex polysaccharides, proteins, lipids and extracellular DNA, collectively called the extracellular polymeric substance (EPS) [1]. Biofilms form when a microbe irreversibly attaches itself to a surface and commences cell division and recruitment of other microorganisms by providing more diverse adhesion sites to the substrate [2]. Being in a biofilm provides microbes with a host of advantages, including, but not limited to: physical protection from the host immune system and antimicrobials/antibiotics, retention of water and tolerance to desiccation, nutrient sorption and storage, high extracellular enzymatic activity, adhesion to the infection site, and cell aggregation leading to coordination of virulence factor expression via quorum sensing [1,3,4]. Particularly troubling to the medical field, it has been estimated that as much as 80% of all human bacterial infections are biofilm-associated, including more than 90% of all chronic wound infections [5,6]. Additionally, the biofilm mode of microbial life is responsible for up to a 1000-fold increase in antibiotic tolerance [7] due to the physical impedance and enzymatic inactivation of the drugs, coupled with lowered metabolic rates in many biofilm-associated cells [8]. Thus, biofilm infections are highly recalcitrant, and are associated with chronic, non-healing infections.

Traditionally, infections have been treated by directly targeting the causative pathogens. However, biofilms change the game by providing microbes with greatly increased protection from antimicrobials, causing the effective concentrations to be elevated to dangerous levels. Therefore, some researchers have switched their focus to anti-biofilm agents that deny the pathogens the protection of the biofilm, thereby increasing the effectiveness of traditional, antimicrobial therapies. One such avenue of research has been the testing of compounds and strategies that lead to a dispersal event: dispersal agents.

**Figure 1:**
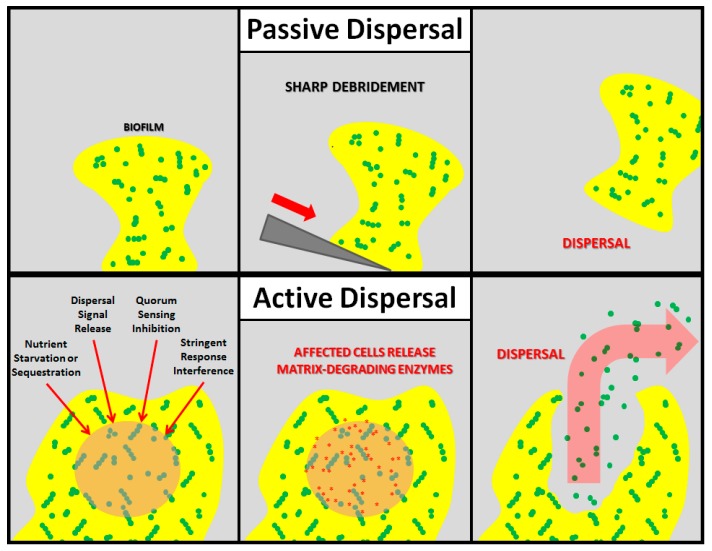
Schematic representations of passive and active biofilm dispersal. In **passive dispersal**, an external force (sharp debridement, in this example) causes the complete or partial destruction of the biofilm. In **active dispersal**, the biofilm microbes respond to an antibiofilm stimulus (nutrient starvation/sequestration, dispersal signal release, quorum sensing inhibition, or stringent response interference, in this example) by actively degrading the matrix, thereby releasing planktonic cells.

Nearly all mature biofilms undergo dispersal, which can be divided into two main subtypes: active and passive, both of which result in the release of planktonic, free-floating cells into the environment (Figure 1). Passive dispersal simply refers to a physical sloughing event brought on by external forces such as fluid and solid shear, and mechanical interventions (e.g., tooth brushing). For example, a biofilm streamer may be torn off of the main mass by the flow of interstitial fluid, or a wound-resident biofilm may be physically debrided by a surgeon. Active dispersal, on the other hand, refers to dispersal events triggered by the biofilm microbes themselves in response to environmental changes such as nutrient starvation, toxic byproducts, bacteriophages, phagocyte challenge, antimicrobial stress, and unfavorable oxygen levels. Thus, active dispersal is a vital stage in the life-cycle of a biofilm that contributes to bacterial survival and disease progression.

Clinically, dispersal can be accomplished by utilizing enzymes, small molecules, or any other means to trigger a massive dispersal event, either passive or active, that releases the biofilm-associated microbes into their more vulnerable, planktonic state. In this review, we will summarize the current state of three major classes of medical biofilm dispersal agents as a therapeutic avenue: enzymes, antibiofilm peptides, and dispersal molecules. It should be noted that this review will be concentrating on molecular methods of biofilm dispersal. However, there are a wide variety of mechanical dispersal methods, such as improved debridement techniques, blue light irradiation, and nonthermal plasma therapy, currently being developed.

## 2. Enzymes

One of the main mechanisms by which bacteria achieve active biofilm dispersal is by the production of extracellular enzymes that act on various structural components of the EPS; namely proteins, extracellular DNA (eDNA), and exopolysaccharides. By targeting that which encloses and protects the microbes, these enzymes facilitate the detachment of cells from the biofilm colony, and their planktonic release into the environment. By isolating and purifying these enzymes, clinicians can theoretically add them exogenously to pre-formed biofilms at elevated concentrations in order to achieve interventional dispersal, making the biofilm-associated microbes more susceptible to the host immune system and antibiotics/antimicrobials. Here, various classes of enzymes that have been investigated for the dispersal of medical biofilms will be reviewed: specifically proteases, deoxyribonucleases, and glycoside hydrolases.

### 2.1. Proteases

Extracellular proteins are a major EPS component that can represent a substantial portion of the biofilm’s dry mass [9,10,11,12]. Exoproteins are crucial for the ability of microbes to maintain and modify the EPS [13,14], and certain proteins, such as DNA-binding proteins (DNABPs), functional amyloids/amyloid-like proteins (FA/ALPs), and other biofilm-associated proteins (Baps), are vital contributors to surface and EPS scaffolding adhesion, and the overall physical stability of the biofilm matrix [10,15]. Thus, enzymatically degrading EPS exoproteins has the potential to cause a massive dispersal event.

A plethora of proteases that contribute to biofilm dispersal have been identified. For example, considering the Gram-positive pathogen *Staphylococcus aureus* alone, ten secreted proteases have been identified, and to date, four of those (V8 serine protease (SspA), two staphopains (SspB and ScpA), and aureolysin (Aur)) have been shown to be involved in biofilm disruption [16,17,18,19,20]. Of those four, Aur, ScpA, and SspB have all been shown to promote dispersal of established *S. aureus* biofilms when they were purified and exogenously added in vitro [20], with Aur being the most effective. Table 1 summarizes many of the proteases that have been shown to have anti-biofilm activity to date.

### 2.2. Deoxyribonucleases

In many biofilms, extracellular DNA (eDNA) functions as a structural scaffolding within the EPS, and can help facilitate bacterial adhesion, aggregation, and horizontal gene transfer [38,39,40,41,42]. Initially, it was assumed that the DNA found within biofilms was merely a remnant of lysed cells, and the first study that showed that eDNA can be a vital, contributing component of bacterial biofilms was done by Whitchurch et al. in 2002 [41]. The authors showed that exogenously added deoxyribonuclease (DNase I) was able to inhibit the formation of *P. aeruginosa* biofilms in vitro without significantly affecting bacterial viability. Additionally, they found that treating established *P. aeruginosa* biofilms up to 60 h with DNase I led to dispersal [41]. This finding triggered a wave of research into targeting eDNA with various DNases as a means to eradicate biofilm infections. Table 2 summarizes many of the DNases that have been shown to have biofilm-disrupting activity to date.

### 2.3. Glycoside Hydrolases

Most biofilms are highly dependent on the presence of secreted extracellular polysaccharides, or exopolysaccharides, as major EPS constituents [1,64,65]. They provide many important functions for the establishment and persistence of biofilms including, but not limited to, structural stability, physical and chemical defense against antimicrobials and the host immune system, adhesion and aggregation of microbial cells, desiccation tolerance, sorption of organic and inorganic compounds, and can provide a carbon source in times of nutrient starvation [1,66,67]. Due to their importance for the establishment and maintenance of biofilm architecture, a significant amount of research into targeting exopolysaccharides with glycoside hydrolases as a means for dispersing biofilms has been performed. Table 3 lists many of the glycoside hydrolases that have exhibited biofilm-disrupting ability.

## 3. Antibiofilm Peptides

In response to the rampant and alarming rise of antibiotic resistance, many researchers have pursued the use of antimicrobial peptides as a novel approach to treating infection. To date, more than 2600 peptides with antimicrobial properties have been discovered, with 2169 of those being antibacterial [68]. These peptides have been isolated from a wide range of sources, including animals, plants, fungi, and bacteria. In higher-order organisms, antimicrobial peptides serve as host defense molecules of the innate immune system, while in simpler organisms, they can be utilized in antagonistic interactions with other microbes for nutrient competition. In recent years, peptides with antibiofilm activity have also been identified and generated, beginning with the discovery that the human cathelicidin, LL-37, is able to inhibit and diminish *P. aeruginosa* biofilms at concentrations far below antimicrobial levels [69].

Unlike a majority of the dispersal agents listed so far in this review, antibiofilm peptides have the added advantage that many are also bactericidal, limiting the need for concurrent treatment with additional antimicrobial agents. Conversely, having bactericidal activity may lead to a higher likelihood of resistance being developed. To date, multiple antibiofilm peptides have been identified, and Table 4 summarizes many that have displayed the ability to disperse established biofilms. It should be noted that, based on the current literature, it cannot be determined that all of these peptides cause dispersal by means other than simply penetrating the EPS and killing the microbes (as is currently understood to be that case for lipopeptide antibiotics, such as colistin and polymyxin b). However, as mentioned above for LL-37, certain peptides cause biofilm destruction at sub-MIC levels, suggesting that they are acting on the EPS, or on the microbe’s ability to form or maintain a biofilm.

## 4. Dispersal Molecules

Other strategies that have been pursued for biofilm eradication include the utilization of molecules that trigger biofilm degradation by various means, such as acting as dispersal signals when recognized by the causative microbes (Table 5; **Dispersal Signals**), physically destabilizing EPS structure (Table 5; **Anti-Matrix Molecules**), or interfering with other, biofilm-sustaining signals (Table 5; **Sequestration Molecules**).

Many key dispersal signals have been identified. For example, endogenously produced nitric oxide (NO) is a highly conserved dispersal mediator that is generated by, and recognized by, an eclectic range of microbes, both prokaryotic and eukaryotic, and it has been brilliantly reviewed by Barraud et al. [88]. Another widely conserved biofilm mediator is the secondary messenger, cyclic di-GMP (c-di-GMP) [89], which has been shown to be important in the establishment and maintenance of biofilms, as well as other key processes, in a plethora of bacterial species. Unlike NO, elevated c-di-GMP levels are almost always associated with increased biofilm production, therefore molecules that bind c-di-GMP, or inhibit diguanylate cyclases, represent potential dispersal agents.

Another class of biofilm dispersal molecules is those that actively target the EPS matrix, also called, ‘anti-matrix molecules.’ Prime examples of this type of molecule are rhamnolipids, which are microbial-synthesized biosurfactants that were first found to be associated with *P. aeruginosa* biofilms [109]. Interestingly, while normal rhamnolipid concentrations are important for the maintenance of mature biofilms, particularly for fluid channel maintenance and cellular migration, elevated levels cause biofilm dispersal for a range of bacterial species [109,110,111,112].

Other dispersal molecules act by binding or interfering with other molecules involved in the production or persistence of biofilm. These ‘sequestration molecules’ may not directly act upon biofilm microbes, but by reducing the levels of important secondary messengers, metabolites, and nutrients, active dispersal can be triggered. For example, BdcA is a protein produced by *E. coli* that binds free c-di-GMP, indirectly inhibiting biofilm by blocking the molecule’s biofilm-producing and biofilm-sustaining cellular pathways [113,114,115].

Research into utilizing molecules such as these to disperse biofilms is extensive, and Table 5 summarizes some of the most prominent, representative examples in the literature. Because of the smaller size of many of these molecules, some that have been shown to be effective against biofilms simply act by penetrating the biofilm and directly killing the microbes. Since such molecules, such as silver or zinc oxide nanoparticles, or chlorhexidine, are not technically dispersal agents, they are beyond the scope of this review and will not be discussed. Additionally, even though anti-biofilm peptides can be classified as dispersal molecules, they were discussed in the previous section.

Lastly, many quorum sensing inhibitors (QSIs) have been shown to be effective against established biofilms by inhibiting the cell-to-cell communication systems that are responsible for the coordination of virulence factors, including biofilm formation. Although these QSIs could be categorized as dispersal agents, the research in this area is extensive and not all quorum sensing systems positively control biofilm formation. For those reasons, QSIs will not be included in this review. For an excellent resource on utilizing QSIs see Brackman and Coenye, 2015 [141].

## 5. Hurdles to Development

With the given breadth of research into the utilization of dispersal agents for the eradication of medical biofilms, there remains a paucity of in vivo studies and clinical trials. In reality, that vast majority of studies have been performed in vitro on monospecies biofilms, and it is extremely difficult to extrapolate these results to complex, multispecies biofilms in living environments. As such, several hurdles exist that have hindered the progress towards the practical application of dispersal agents in healthcare, such as potential host-toxicity, especially when considering the utilization of proteases and other enzymes that may cause collateral damage. Also, inhibitory interactions within the host environment, such as proteolytic degradation or small-molecule inhibition of therapeutic agents, may further complicate the transition to clinical application. Further, while dispersing microbes from biofilms may improve therapeutic outcomes by increasing access by antibiotics/antimicrobials and host immune cells, it has been suggested that this may not be the best approach for fungal species. That is, since fungal biofilm dispersal is often initiated from the hyphal layers, perhaps it would be advantageous to instead *prevent* dispersal in fungal biofilms so as to contain the infection and avert the dissemination of the disease [142].

Finally, for those agents that have no inherent antimicrobial activity, it is unknown whether triggering a massive dispersal event within a host will effectively overload the immune system with a planktonic cell burden, leading to spread and sepsis. It has even been shown that dispersed cells may in fact be *more* virulent than not only biofilm cells, but also regular planktonic cells [143,144]. Thus, concurrent treatment with traditional antimicrobial agents and/or therapies may be necessary. Despite these complications, research into biofilm dispersal is a booming, promising field, and the likelihood of applicable therapies coming into use in the near future appears high, especially when considering the alarming rise of antibiotic resistance, and the resulting, growing need for alternative treatment strategies.

## 6. Conclusions

The rise of antibiotic resistance has led to a decrease in the efficacy of traditional treatments for the elimination of biofilm infections. Because of the up to 1000-fold increase in antibiotic tolerance of biofilm-embedded pathogens [7], and the fact that as many as 80% of all human bacterial infections are biofilm-associated [6], researchers and clinicians have begun concentrating their efforts on coupling biofilm destruction with traditional antimicrobial therapy. However, current biofilm removal practices are purely mechanical, and, as such, it is extremely difficult to eradicate the entire infection, leading to recurrence. To address this, clinicians couple mechanical biofilm removal, such as sharp or hydrosurgical debridement, with antibiotics/antimicrobials. For example, the current gold standard for the treatment of chronic wound infections is repeated sharp debridement followed by topical administration of antimicrobials or other therapies [5,145,146]. Nonetheless, even with these interventions, these recalcitrant wounds all too often fail to heal, and antimicrobial resistances fortify. Thus, novel biofilm dispersal strategies that can more effectively release biofilm-associated microbes from the protection of the EPS could serve to bolster the arsenal of anti-biofilm therapeutics. Dispersal agents that can target the EPS on a molecular scale, or cause the microbes themselves to actively degrade their own biofilms, may represent the next logical step towards total eradication of biofilm-afforded protection of infectious microorganisms. In this review, we examined the current state of the three main avenues of research into molecular biofilm dispersal agents: matrix-degrading enzymes, antibiofilm peptides, and dispersal molecules. We find that, despite the plethora of potential agents, few clinical trials, or even in vivo studies, have been performed. Thus, even though the future use of dispersal agents for the treatment of medical biofilms looks promising, progress needs to be made on translating the work to the patient care setting.

## Figures and Tables

**Table 1:** Proteases that Disperse Biofilms.

Enzyme	Summary	References
Aureolysin (Aur)	A staphylococcal metalloprotease that has been shown to disrupt *S. aureus* biofilms by degrading Bap and clumping factor b.	[17,20]
LapG Protease	A protease produced by *Pseudomonas putida* that has been shown to trigger dispersal through modification of the outer membrane-associated, exopolysaccharide-binding protein, LapA.	[21]
Proteinase K	A highly reactive and stable serine protease that exhibits a broad range of cleavage by targeting peptide bonds adjacent to the carboxylic group of aliphatic and aromatic amino acids. It is active against the biofilms produced by a range of bacteria, including *S. aureus*, *Listeria monocytogenes*, *Staphylococcus lugdunensis, Staphylococcus heamolyticus*, *Gardnerella vaginalis*, and *Escherichia coli*, *Heamophilus influenza*, and *Bdellovibrio bacteriovorus*.	[22,23,24,25,26,27,28,29]
Spl Proteases	A group of six Staphylococcal serine proteases that have been shown to be involved in *S. aureus* biofilm dispersal, possibly by cleaving the cell wall-associated protein, EbpS.	[30,31]
Staphopain A (ScpA), Staphopain B (SspB)	Staphylococcal cysteine proteases that have been shown to disperse *S. aureus* biofilms through degradation of (an) unknown target(s).	[19,20]
Streptococcal Cysteine Protease (SpeB)	A *Streptococcus pyogenes* cysteine protease that is historically known to be involved in immune evasion by the pathogen, owing to its cleavage of host immune molecules, as well as tissue invasion by way of host ECM degradation. However, SpeB has more recently been shown to be involved in dispersal of in vivo *S. pyogenes* biofilms via hydrolysis of surface proteins M and F1, which are hypothesized to be involved in microcolony formation.	[32,33]
Surface-protein-releasing enzyme (SPRE)	An endogenous Streptococcal protease that has been shown to cause *Streptococcus mutans* monolayer biofilm detachment from a colonized surface via the release of the surface protein, antigen P1.	[34]
Trypsin	A pancreatic serine protease that cleaves peptides at the carboxyl side of lysine or arginine. It is active against biofilms made by multiple bacterial species, including *Pseudomonas aeruginosa*, *Streptococcus mitis*, *Actinomyces radicidentis*, *Staphylococcus epidermidis*, and *Gardnerella vaginalis*.	[25,26,35,36]
V8 Serine Protease (SspA)	A staphylococcal serine protease that degrades fibronectin binding proteins and Bap in *S. aureus* biofilms.	[17,37]

**Table 2:** DNases that Disperse Biofilms.

Enzyme	Summary	References
DNase I	A pancreatic DNase that has been shown to deconstruct the established biofilms of a wide range of microbes, including *P. aeruginosa*, *Vibrio cholerae*, *E. coli*, *S. pyogenes*, *Klebsiella pneumoniae*, *Acinetobacter baumannii*, *Aggregatibacter actinomycetemcomitans*, *Shewanella oneidensis*, *S. heamolyticus*, *Bordetella pertussis*, *Bordetella bronchiseptica*, *Campylobacter jejuni*, *H. influenza*, *B. bacteriovorus*, *S. aureus*, *Enterococcus faecalis*, *Listeria monocytogenes*, *Candida albicans*, and *Aspergillus fumigatus.*	[27,28,29,41,43,44,45,46,47,48,49,50,51,52,53,54,55,56,57]
DNase 1L2	A human DNase found in keratinocytes that has been shown to degrade the established biofilms of *P. aeruginosa* and *S. aureus*.	[58]
Dornase alpha	A highly purified form of recombinant human DNase I (rhDNase I) that has been shown to be effective against the established biofilms of *S. aureus* and *Streptococcus pneumoniae*.	[59,60]
λ Exonuclease	A viral DNase that disrupts established *V. cholerae* biofilms.	[43]
NucB	A bacterial DNase produced by the marine bacterium, *Bacillus licheniformis*, which has been show able to degrade the established biofilms of multiple bacterial species, including *B. licheniformis*, *S. aureus*, *S. epidermidis*, *Staphylococcus salivarius*, *Staphylococcus constellatus*, *S. Staphylococcus lugdunesis*, *Staphylococcus anginosus*, *E. coli*, *Streptococcus intermedius*, *Micrococcus luteus*, and *Bacillus subtilis*.	[61,62,63]
Streptodornase	A streptococcal DNase that disrupts the established biofilms of *P. aeruginosa*.	[56]

**Table 3:** Glycoside Hydrolases that Disperse Biofilms.

Enzyme	Summary	References
Alginate lyase	A glycoside hydrolase that that degrades the exopolysaccharide, alginate, common in mucoid *P. aeruginosa* biofilms, causing bacterial cell dispersal and increasing antibiotic efficacy and phagocytosis.	[70,71,72,73]
α-amylase	A glycoside hydrolase that hydrolyzes α(1,4) glycosidic linkages and is derived from multiple sources, such as certain microbes and the mammalian pancreas. It has exhibited dispersal of mature biofilms formed by *V. cholerae*, *S. aureus* and *P. aeruginosa*.	[74,75,76,77]
α-mannosidase	An acid hydrolase that has been shown to disrupt *P. aeruginosa* biofilms.	[35]
β-mannosidase	A glycoside hydrolase that targets β(1,4)-linked terminal mannose residues, and disrupts *P. aeruginosa* biofilms.	[35]
Cellulase	A glycoside hydrolase produced by multiple microbes that hydrolyzes the β(1,4) glycosidic linkage. It has been shown to cause the dispersal of *S. aureus* and *P. aeruginosa* biofilms.	[77]
Dispersin B	A glycoside hydrolase produced by the bacterium, *A. actinomycetemcomitans*, that has been shown to degrade the polysaccharide, poly(1,6)-*N*-acetyl-d-glucosamine (PNAG), by hydrolyzing β(1,6) glycosidic linkages. This enzyme has been effective against the biofilms made by multiple bacteria, including *S. aureus*, *A. actinomycetemcomitans*, *S. epidermidis*, *A. baumannii*, *K. pneumoniae*, *E. coli*, *Burkholderia* spp., *A. pleuropneumoniae*, *Yersinia pestis* and *Pseudomonas fluorescens*.	[57,78,79,80,81,82,83,84]
Hyaluronidase	An enzyme that cleaves hyaluronic acid (HA), which has been found to be incorporated into the biofilms made by multiple pathogens, including *S. aureus*, and *S. intermedius* in vivo. When utilized against HA-containing biofilms, dispersal has been observed.	[85,86]
PelAh	A glycoside hydrolase that disrupts the *P. aeruginosa* polysaccharide, Pel, causing dispersal of mature biofilms.	[87]
PslGH	A glycoside hydrolase that disrupts the *P. aeruginosa* polysaccharide, Psl, causing dispersal of mature biofilms.	[87]

**Table 4:** Antibiofilm Peptides that Disperse Biofilms.

Peptide	Summary	References
1018	A synthetic, modified form of the cationic antimicrobial peptide bactenecin, which triggers the degradation of the (p)ppGpp bacterial stringent response signal. This peptide has been shown to be effective at disrupting the established biofilms of *P. aeruginosa*, *E. coli*, *A. baumannii*, *K. pneumoniae*, *S. aureus*, *Salmonella Typhimurium*, and *Burkholderia cenocepacia*.	[90,91]
1037	A 9-amino-acid, synthetic, cationic peptide derived from the human cathelicidin LL-37, which has demonstrated efficacy against biofilms made by *P. aeruginosa*, *B. cenocepacia*, and *L. monocytogenes*.	[92]
17BIPHE2	A 17-amino-acid derivative of the human cathelicidin, LL-37, that has had exhibited efficacy in disrupting *S. aureus* biofilms.	[93]
Bac8c	A 12-amino-acid, synthetic peptide modified from bactenecin that has exhibited efficacy against *S. mutans* biofilms.	[94]
Battacin	A native, cyclic lipopeptide produced by *Paneibacillus tianmunesis*, whose synthetic derivatives, especially lipopeptide 17, have been shown to degrade *P. aeruginosa* and *S. aureus* biofilms.	[95]
BMAP-27	A synthetic, bovine cathelicidin-derived peptide that has exhibited efficacy against *S. aureus*, *P. aeruginosa*, and *Stenotrophomonas maltophilia* biofilms.	[96]
BMAP-28	A synthetic, bovine cathelicidin-derived peptide that has exhibited efficacy against *S. aureus*, *P. aeruginosa*, and *S. maltophilia* biofilms.	[96,97]
CAMA	A hybrid, synthetic peptide that combines amino acid sequences from the silk moth peptide, cecropin-A, and the bee venom peptide, melittin. It has exhibited the ability to degrade *P. aeruginosa* and *S. aureus* biofilms.	[98,99]
DJK-5	A synthetic, D-enantiomeric, protease-resistant peptide that works, in part, by degrading the (p)ppGpp bacterial stringent response signal. It has been shown to be effective at disrupting *P. aeruginosa*, *A. baumannii*, *Salmonella enterica* and *K. pneumoniae* biofilms.	[100]
DJK-6	A synthetic, D-enantiomeric, protease-resistant peptide that works, in part, by degrading the (p)ppGpp bacterial stringent response signal. It has been shown to be effective at *P. aeruginosa*, *A. baumannii*, *S. enterica* and *K. pneumoniae* biofilms.	[100,101]
GF-17	A 17-amino-acid derivative of the human cathelicidin, LL-37, that has exhibited efficacy in disrupting *S. aureus* biofilms.	[93]
LL-31	A synthetic fragment of the human cathelicidin, LL-37, in which the last 6 amino acid residues are removed. The peptide has been shown to disrupt *P. aeruginosa* biofilms.	[102]
LL-37	A 37-amino-acid, native human cathelicidin that has been shown to disrupt *A. baumannii* and *P. aeruginosa* biofilms.	[69,103,104,105]
LL7-31	A synthetic fragment of the human cathelicidin, LL-37, in which the first 6, and last 6, amino acid residues are removed. The peptide has been shown to disrupt *P. aeruginosa* biofilms.	[102]
LL7-37	A synthetic fragment of the human cathelicidin, LL-37, in which the first 6 amino acid residues are removed. The peptide has been shown to disrupt *P. aeruginosa* biofilms.	[102]
Melittin	A native, 26-amino-acid, haemolytic peptide, isolated from the venom of the European honey bee, *Apis mellifer*. The peptide has been efficacious against *P. aeruginosa*, *E. coli* and *K. pneumonia* biofilms.	[106]
P10	A synthetic, 24-amino-acid peptide derived from the P60.4AC (which itself is a derivative of the human cathelicidin, LL-37) that has been shown to degrade *S. aureus* biofilms.	[107]
P60.4Ac	A synthetic, 24-amino-acid peptide derived from the human cathelicidin, LL-37, which has been shown to be effective at degrading *S. aureus* biofilms.	[107,108]
SMAP-29	A synthetic, sheep cathelicidin-derived peptide that has exhibited efficacy against *S. aureus*, *P. aeruginosa*, and *Stenotrophomonas maltophilia* biofilms.	[96]

**Table 5:** Biofilm Dispersing Molecules.

Molecule	Summary	References
**Dispersal Signals**		
*Cis*-2-decenoic acid (CDA)	A type of fatty acid cross-kingdom signaling molecule, also known as a diffusible signal factor (DSF), which was originally found to be produced by *P. aeruginosa*. This particular DSF has been shown to trigger the dispersal of *P. aeruginosa*, *E. coli*, *K. pneumoniae*, *P. mirabilis, S. pyogenes, B. subtilis, S. aureus, C. albicans*, *S. enterica*, and *S. mutans* biofilms. It should be noted that other DSF’s, such as *Burkholderia* diffusible signal factor (BDSF) [116] and *Xanthomonas* diffusible signal factor (XDSF) [117], have been isolated and exhibit similar inductions of dispersal events.	[118,119,120,121]
Nitric oxide	An endogenously produced dispersal signal that is highly conserved across a wide range of microbial species. It has been shown to be involved in the dispersal of biofilms formed by *P. aeruginosa, E. coli*, *Fusobacterium nucleatum*, *Serratia marcescens*, *V. cholerae*, *B. licheniformis*, *Shewanella woodyi*, *N. gonorrhoeae*, *Pseudoalteromonas*, *Vibrio fischeri*, *S. aureus*, *B. subtilis*, *Legionella pneumophila*, *Nitrosomonas europaea*, *P. putida*, *C. albicans*, *Candida tropicalis*, and *Ulva linza*.	[88]
**Anti-Matrix Molecules**		
Chitosan	A polycationic macromolecule derived from the polysaccharide, chitin that has been shown to penetrate and possibly disrupt *Cryptococcus neoformans*, *L. monocytogenes*, *P. fluorescens*, *Bacillus cereus*, *S. enterica*, *C. albicans*, and *P. aeruginosa* biofilms. It is important to note that it has not been proven that chitosan has any direct effect on the biofilm matrix, and it is possible that the molecule achieves biofilm disruption by penetrating the matrix and acting on the microbes themselves.	[122,123,124,125,126]
d-amino acids	d-isoforms of certain amino acids, including d-Leu, d-Met, d-Trp, d-Tyr, and d-Phe have been shown to cause the disassembly of biofilms though to multiple, hypothesized mechanisms, including (1) inhibition of genes involved in EPS production; and (2) incorporation of d-amino acids into the bacterial cell wall, resulting in the loss of cell-surface fibers vital to biofilm formation. d-amino acids have exhibited efficacy against *S. aureus*, *P. aeruginosa*, and *B. subtilis* biofilms.	[127,128,129,130]
Rhamnolipids	A microbial-produced surfactant that, at normal levels, is important for biofilm maturation in the form of fluid channel maintenance and cellular migration. At elevated levels, however, these rhamnolipids have been shown to trigger the dispersal of *P. aeruginosa*, *E. coli*, *S. aureus, B. subtilis*, *M. luteus*, and *Yarrowia lipolytica* biofilms.	[109,110,111,112]
Urea	An amide that is theorized to break down biofilms by disrupting the hydrogen bonds that are vital for EPS mechanical stability. The compound has exhibited dispersal ability against *S. epidermidis*, *P. aeruginosa* and *K. pneumoniae* biofilms.	[131,132]
**Sequestration Molecules**		
BdcA	A protein that directly reduces unbound c-di-GMP concentrations by binding, but not degrading, the molecules, causing biofilm-related cellular mechanisms not to be activated. BdcA has been shown to cause the dispersal of *E. coli*, *P. aeruginosa*, *P. fluorescens*, and *Rhizobium meliloti* biofilms.	[113,114,115]
EDTA	Ethylenediaminetetraacetic acid (EDTA) is a metal-ion chelator that can sequester integral, EPS-matrix-stabilizing ions, and triggers dispersal in *P. aeruginosa*, *H. influenzae*, *S. epidermidis*, *C. tropicalis*, and *E. faecalis* biofilms.	[133,134,135,136,137,138]
Lactoferrin	An iron-binding protein from the innate immune system that is found in a variety of bodily fluids. By chelating iron, an essential bacterial nutrient and global regulator of a variety of functions, including biofilm development and growth, lactoferrin can trigger active dispersal. It has been shown to be effective against *P. aeruginosa*, *E. coli*, *S. aureus*, *E. faecalis* and *S. epidermidis* biofilms.	[139,140]
